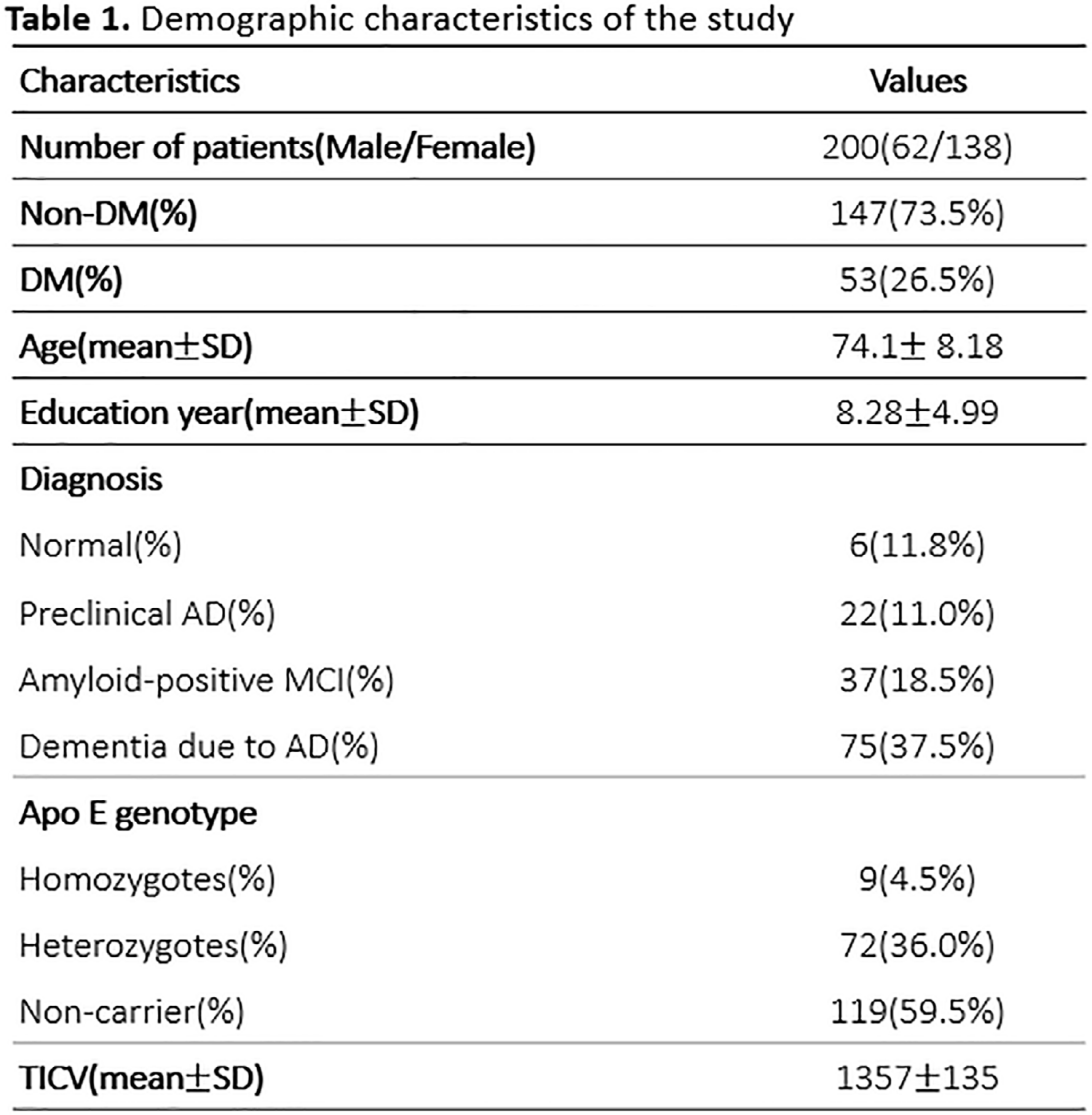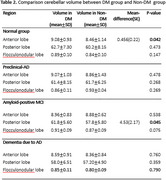# Diabetes mellitus‐induced differential cerebellar volume reduction across Alzheimer's disease trajectory

**DOI:** 10.1002/alz70856_097584

**Published:** 2025-12-24

**Authors:** Suhyung Kim, Sheng‐Min Wang, Dong Woo Kang, Sunghwan Kim, Hyun Kook Lim, Yoo Hyun Um

**Affiliations:** ^1^ St. Vincent's Hospital, the Catholic University of Korea, Suwon, Gyeonggi‐do, Korea, Republic of (South); ^2^ College of Medicine, Yeouido St. Mary's Hospital, The Catholic University of Korea, seoul, seoul, Korea, Republic of (South); ^3^ College of Medicine, Seoul St. Mary's Hospital, The Catholic University of Korea, Seoul, Seoul, Korea, Republic of (South); ^4^ Yeouido St. Mary's Hospital, College of Medicine, The Catholic University of Korea, Seoul, Korea, Korea, Republic of (South); ^5^ Yeouido St. Mary's Hospital, College of Medicine, The Catholic University of Korea, Seoul, Korea, Republic of (South); ^6^ St. Vincent' Hospital, College of Medicine, The Catholic University of Korea, Suwon, Gyeonggi‐do, Korea, Republic of (South)

## Abstract

**Background:**

Alzheimer's disease (AD) and diabetes mellitus (DM) are associated with neurodegeneration, yet their combined effects on the cerebellum remain unclear. While prior studies highlight cortical and hippocampal atrophy in AD, the cerebellum's role has been largely overlooked. This study examines DM's impact on cerebellar volume across AD stages, including normal cognition, preclinical AD, and amyloid‐positive mild cognitive impairment (MCI).

**Method:**

A total of 134 patients with AD pathology and 66 normal controls were classified into normal cognition (66), preclinical AD (22), amyloid‐positive MCI (37), and AD dementia (75), stratified by DM status. Cerebellar volumes of the anterior, posterior, and flocculonodular lobes were analyzed. Cognitive grouping was based on the Consortium to Establish a Registry for Alzheimer's Disease‐Korean version (CERAD‐K) assessments, and amyloid positivity was determined via [^18F] flutemetamol PET imaging. MRI data were processed using voxel‐based morphometry (VBM) with the CAT12 toolbox, and cerebellar segmentation was performed using the Spatially Unbiased Infratentorial Template (SUIT) atlas. ANCOVA, adjusted for age, sex, and total cranial volume, assessed cerebellar volume differences across groups.

**Results:**

DM was associated with reduced anterior lobe volume in normal cognition (*p* = 0.042) and exacerbated posterior lobe atrophy in amyloid‐positive MCI (*p* = 0.045, Bonferroni‐corrected). No significant changes were observed in preclinical AD.

**Conclusion:**

DM influences cerebellar volume differently across the AD spectrum. Anterior lobe atrophy in normal cognition suggests early cerebellar vulnerability to DM, while posterior lobe atrophy in amyloid‐positive MCI indicates accelerated degeneration in advanced amyloid pathology. The absence of significant changes in preclinical AD may reflect compensatory mechanisms. These findings highlight the complex interaction between DM and AD, with DM potentially exacerbating cerebellar degeneration in later disease stages.